# Better Identification of Cognitive Decline With Interleukin-2 Than With Amyloid and Tau Protein Biomarkers in Amnestic Mild Cognitive Impairment

**DOI:** 10.3389/fnagi.2021.670115

**Published:** 2021-05-28

**Authors:** Chih-Sung Liang, Chia-Lin Tsai, Guan-Yu Lin, Jiunn-Tay Lee, Yu-Kai Lin, Che-Sheng Chu, Yueh-Feng Sung, Chia-Kuang Tsai, Ta-Chuan Yeh, Hsuan-Te Chu, Ming-Wei Su, Fu-Chi Yang

**Affiliations:** ^1^Department of Psychiatry, Beitou Branch, Tri-Service General Hospital, National Defense Medical Center, Taipei, Taiwan; ^2^Graduate Institute of Medical Sciences, National Defense Medical Center, Taipei, Taiwan; ^3^Department of Neurology, Tri-Service General Hospital, National Defense Medical Center, Taipei, Taiwan; ^4^Department of Psychiatry, Kaohsiung Veterans General Hospital, Kaohsiung, Taiwan; ^5^Center for Geriatric and Gerontology, Kaohsiung Veterans General Hospital, Kaohsiung, Taiwan; ^6^Department of Psychiatry, Tri-Service General Hospital, National Defense Medical Center, Taipei, Taiwan; ^7^Institute of Biomedical Sciences, Academia Sinica, Taipei, Taiwan

**Keywords:** predictive biomarkers, Alzheimer’s disease, amnestic mild cognitive impairment, interleukin-2, beta amyloid, tau protein

## Abstract

The rate of cognitive decline among patients with amnestic mild cognitive impairment (aMCI) varies, and it is thus crucial to accurately predict the probability of cognitive deterioration in patients with MCI. We compared the potential of cytokines with amyloid beta (Aβ) and tau biomarkers for predicting cognitive decline in patients with aMCI or Alzheimer’s disease (AD). All participants (controls, aMCI, and AD patients) underwent plasma biomarker examinations for Aβ_1–40_, Aβ_1–42_, total tau (t-tau), tau phosphorylated at threonine 181 [*p*-Tau181]), and 29 cytokines and baseline cognitive tests, including Mini-Mental State Examination (MMSE). The correlation between biomarker levels and annual MMSE change during the follow-up was examined. Receiver operating characteristic (ROC) curve analysis was performed to determine whether the statistically significant plasma biomarkers could identify cognitive decline. Higher baseline levels of IL-2, sCD40L, IL-8, and VEGF were associated with a lower annual cognitive decline in the aMCI group, and higher baseline levels of Aβ_1–40_, IFNγ, IL-5, IL-17A, IL-25, and FGF were associated with a rapid annual cognitive decline in the AD group. IL-2 had a high discriminatory capacity for identifying cognitive decline, with an area under curve (AUC) of 85.7% in the aMCI group, and the AUC was slightly increased when combining IL-2 with Aβ or tau biomarkers. However, none of the biomarkers had a satisfactory discriminatory capacity in the AD group. IL-2 may have a better discriminatory capacity for identifying cognitive decline than Aβ and tau biomarkers in patients with aMCI.

## Introduction

Amnestic mild cognitive impairment (aMCI) is a heterogenous, symptomatic pre-dementia condition that represents a transitional phase between normal ageing and dementia (and often Alzheimer’s disease [AD]) (Petersen, 2011). The rate of cognitive decline among patients with aMCI varies, some patients progress to dementia, while others remain stable or even revert to normal cognition (Petersen, 2011). Depending on the population studied, the annual rate of conversion from aMCI to dementia ranges from less than 5–20% (Langa and Levine, 2014). However, many patients may not progress to dementia when followed up after 10 years (Mitchell and Shiri-Feshki, 2009), implying that patients diagnosed with aMCI may live with uncertainty for a long time (Langa and Levine, 2014). Therefore, it is crucial to accurately identify and predict the probability of cognitive deterioration in patients with aMCI.

In recent decades, significant effort has been devoted to diagnosing aMCI or dementia early using a variety of biomarkers. An accurate diagnosis and prognosis of aMCI is important for patients and their families to determine appropriate methods of care and future plans. The commonly suggested biomarkers are: an apolipoprotein E ε4 carrier status; atrophy on structural magnetic resonance imaging; hypometabolism on fludeoxyglucose F 18-positron emission tomography; and levels of several cerebrospinal fluid (CSF) biomarkers, such as amyloid β 1-42 peptide (Aβ_1–42_), total tau, and tau phosphorylated at threonine 181 (*p*-Tau181) (Petersen et al., 2009). In recent years, plasma biomarkers have shown their potential for predicting the burden of Aβ in the brain and identifying rapid cognitive decline in patients with aMCI (Nakamura et al., 2018; Lue et al., 2019; Tsai et al., 2019, 2020). For example, a large cohort study reported that longitudinal changes of plasma *p*-Tau181 were associated with cognitive decline and neurodegeneration in the brain (Moscoso et al., 2021). Undoubtedly, biomarkers enable broader clinical access and efficient screening in individuals with risk factors of cognitive decline.

Although AD is characterized by a complex interplay between abnormal Aβ and tau proteins, evidence has suggested that the innate immune system-mediated inflammation drives the AD pathogenesis (Heneka et al., 2015; Heppner et al., 2015). Neuroinflammation may trigger a vicious cycle of microglial activation, release of pro-inflammatory factors, and neuronal damage in the preclinical stage of AD (Heneka et al., 2015; Heppner et al., 2015). Clinical studies have found that patients with aMCI or AD have significantly altered inflammatory biomarkers, such as interleukin (IL)-6 and IL-10 in both the peripheral and CSF (King et al., 2018; Shen et al., 2019). Furthermore, the peripheral levels of inflammatory biomarkers were found to be associated with future cognitive decline in patients with aMCI or AD (Bradburn et al., 2017; Morgan et al., 2019; Liang et al., 2020). For example, a cohort study reported that plasma levels of complement factor B and factor H were associated with aMCI progression to AD (Morgan et al., 2019). Another study reported plasma IL-33 expression was associated with cognitive preservation among patients with aMCI or AD (Liang et al., 2020). However, to date, no studies have compared the potential of cytokines versus Aβ- or tau-related biomarkers for predicting cognitive decline among patients with aMCI or AD.

The aim of this study was to compare the potential of 29 cytokines with Aβ and tau biomarkers for predicting cognitive decline among patients with aMCI or AD. We hypothesized that cytokines may have a better discriminatory ability than Aβ and tau biomarkers for predicting cognitive decline.

## Methods

### Subjects and Study Design

The protocol was approved by the Institutional Review Board for the Protection of Human Subjects at the Tri-Service General Hospital (TSGHIRB 1-107-05-111). Written informed consent was obtained from all participants. Between January 2015 and December 2019, participants were recruited from the memory clinic of the Tri-Service General Hospital of the National Defense Medical Center, Taiwan. Individuals were eligible if they were aged 60 years or older and had negative findings on physical and neurological examinations, laboratory tests (assessment of creatinine, fasting blood sugar, free-thyroxine 4, high-sensitivity thyroid-stimulating hormone, vitamin B12, and folic acid; serologic test for syphilis; and routine blood tests) and neuroimaging examinations (brain computed tomography or magnetic resonance imaging).

Individuals were excluded if they had: (a) a history of major or uncontrolled medical conditions, such as heart failure, sepsis, liver cirrhosis, renal failure, chronic obstructive pulmonary disease, poorly controlled diabetes (Hemoglobin A1c > 8.5), myocardial infarction, or malignancy; (b) substance abuse; (c) a history of major neurological disorders, such as stroke or Parkinson’s disease; (d) a score >9 in the short-form Geriatric Depression Scale (GDS-S) or >3 for the modified Rankin Scale; and (e) a history of major psychiatric conditions that can impair cognition, such as major depressive disorder, bipolar disorder, or schizophrenia.

On recruitment, participants underwent the following examinations: Mini-Mental Status Examination (MMSE); Clinical Dementia Rating (CDR); short-form GDS-S; verbal fluency test; Hopkins Verbal Learning Test (HVLT); forward and backward digit span; Trail Making Test, Part A; Modified Boston Naming Test; and Hachinski Ischemia Scale (HIS). The second MMSE was conducted after the 1-year follow-up.

Participants were classified into the control, aMCI, and AD groups based on the results of HVLT, MMSE, and CDR examinations, as well as the recommendations from the National Institute on Aging and Alzheimer’s Association (NIA-AA) workgroups on diagnostic guidelines for AD and aMCI due to AD (Albert et al., 2011; McKhann et al., 2011). A diagnosis of AD was made if patients satisfied the following criteria: (a) NIA-AA criteria (McKhann et al., 2011); (b) CDR ≥ 0.5; (c) MMSE ≤ 26 (middle school), MMSE score ≤ 22 (primary school), or MMSE score ≤ 19 (illiteracy); (d) HIS score ≤ 3; and (e) HVLT score ≤ 19 (Hogervorst et al., 2014). aMCI was diagnosed if patients satisfied the following criteria: (a) NIA-AA criteria (Albert et al., 2011); (b) CDR = 0.5; (c) MMSE score > 26 (middle school), MMSE score > 22 (primary school), or MMSE score > 19 (illiteracy); (d) HIS score ≤ 3; and (e) HVLT score ≤ 22 (Hogervorst et al., 2014). Healthy controls were required to satisfy the following criteria: (a) no active neurological or psychiatric disorders; (b) no psychotropic drugs; (c) MMSE score > 26 (middle school), MMSE score > 22 (primary school), or MMSE score > 19 (illiteracy); and (d) CDR score = 0.

### Plasma Preparation

Fasting blood was collected in 9-mL K3-EDTA tubes (455036, Greiner Bio-one GmbH, Kremsmünster, Austria), which were gently inverted three times immediately following blood collection. Blood samples were then centrifuged at 2,300 *g* for 10 min (4°C) using a swing-out (bucket) rotor (5202R, Eppendorf, Hamburg, Germany). Each 0.4-mL plasma sample was transferred to a fresh 2.0-mL tube (CryzoTraq, Ziath, Cambridge, United Kingdom). All plasma samples were stored in 0.5-ml aliquots within 8 h of blood collection at −80°C until further use.

### Assessment of Plasma Aβ and Tau Levels

Immunomagnetic reduction (IMR), an ultra-sensitive analytical assay, can reliably assay ultra-low concentrations of Aβ and tau biomarkers, including Aβ_1–40_, Aβ_1–42_, total tau (t-Tau), and *p*-Tau181 (Tsai et al., 2019). The levels of plasma Aβ_1–40_, Aβ_1–42_, t-Tau, and *p*-Tau181 were assessed using IMR kits (MF-AB0-0060, MF-AB2-0060, MF-TAU-0060, and MF-PT1-0060, MagQu Co., New Taipei City, Taiwan). For each assay, 40 μL (Aβ_1–40_, t-Tau, and *p*-Tau181) or 60 μL (Aβ_1–42_) of plasma was mixed with 80 or 60 μL of reagent, respectively. Each reported biomarker concentration represented the average of 2 duplicated measurements. An IMR analyzer (XacPro-S, MagQu Co., New Taipei City, Taiwan) was used for all assays. The measured biomarker concentrations ranged from 0.17 to 1,000 pg/mL for Aβ_1–40_, 0.77 to 30,000 pg/mL for Aβ_1–42_, 0.026 to 3,000 pg/mL for t-Tau, and 0.0196 to 1,000 pg/mL for *p*-Tau181. Intra-assay or inter-assay coefficients of variations using IMR ranged from 7 to 10% and from 10 to 15% for high-concentration and low-concentration quality control samples of Aβ_1–40_, Aβ_1–42_, t-Tau, or *p*-Tau181, respectively. For each biomarker, two batches of reagents were used, and the quality of each batch of reagents was well-controlled by monitoring the particle size, particle concentration, and bioactivity. The variation in these reagent properties between batches was lower than 10%.

### Measurement of Plasma Cytokines

A multiplex bead array assay was used to examine plasma cytokines levels. The detailed procedures for detection of soluble cytokines using the multiplex bead array assays have been previously reported (Ho et al., 2015, 2017). We examined 29 cytokines using a customized human cytokine magnetic bead panel (Bio-Rad; Yu-Shing Biotech., Ltd, Taipei, Taiwan) according to the manufacturer’s instructions (Bio-Rad; Genmall Biotechnology Co., LTD., Taipei, Taiwan). The examined cytokines were IL-1β, IL-1 receptor antagonist (IL-1RA), IL-2, IL-4, IL-5, IL-6, IL-7, IL-8, IL-9, IL-10, IL-13, IL-17A, IL-23, IL-25, IL-31, interferon-gamma (IFNγ), tumor necrosis factor-alpha (TNFα), soluble CD40 ligand (sCD40L), IFNγ-induced protein 10 (IP10), monocyte chemoattractant protein 1 (MCP1), macrophage inflammatory protein 1-alpha (MIP1α), MIP1β, regulated upon activation, normal T cell expressed and secreted (RANTES), eotaxin, fibroblast growth factor (FGF), granulocyte colony-stimulating factor (GCSF), Granulocyte-macrophage colony-stimulating factor (GM-CSF), platelet-derived growth factor-BB (PDGF-BB), and vascular endothelial growth factor (VEGF). The median fluorescence intensities were assessed using a Bio-Plex 200 instrument (Bio-Rad) with Bio-Plex Manager software version 6.0 (Bio-Rad). Study samples were assessed in duplicates and the duplicate measurements were averaged for statistical analysis. Standard curves were created from duplicate values and all samples were analyzed as single determinants. All analyses were performed in one batch using kits from the same production lot.

### Statistical Analyses

Group differences (aMCI vs controls and AD vs controls) in categorical variables were examined by using the Fisher’s exact test. Group differences in continuous variables were examined by using the independent sample t-test or Mann–Whitney *U*-test. The temporal ordering (controls, aMCI, AD) of the cognitive tests, Aβ and tau biomarkers, and cytokines were examined by using the P trend analysis. The association between plasma biomarkers (including Aβ, tau, and cytokines biomarkers) and annual change in MMSE score in the aMCI and AD groups was assessed using partial correlation with adjustment for age, education level, body mass index, and apolipoprotein E (APOE) genotype (i.e., the proportion of individuals who carry the APOE ε4 allele). Receiver operating characteristic (ROC) curve analysis was performed for the statistically significant plasma biomarkers in the partial correlation analysis. Using cognitive decline (a change in the annual MMSE score ≥ 2) as the outcome, the utility of individual plasma biomarkers to identify the outcome was evaluated using the area under the ROC curve (AUC). The highest AUC value of the cytokine was combined with each Aβ and tau biomarker for a combined AUC analysis. The 95% confidence interval (CI) for the AUC was calculated using the DeLong’s test. All tests were 2-tailed and *P* < 0.05 was considered statistically significant. No adjustment of multiple testing (multiplicity) was performed in this study. Data analyses were conducted using SPSS 25 (IBM SPSS Inc, Chicago, IL, United States).

## Results

### Patient Demographics and Plasma Biomarkers

A total of 91 participants were included (aMCI = 51, AD = 28, controls = 12) (Table 1). Patients in the AD (78.3 ± 8.8 years) and aMCI groups (75.6 ± 8.6 years) were older than those in the control group (66.3 ± 5.9 years). All the cognitive tests showed a linear trend in the control, aMCI, and AD groups, and both the aMCI and AD groups had poorer cognitive test results than the control group. In terms of Aβ and tau biomarkers, five biomarkers showed a linear trend in the three groups, including t-Tau, *p*-Tua181, Aβ_1–42_, Aβ_1–42_ x t-Tau, and Aβ_1–42_ x *p*-Tau181 levels. Compared with the control group, the AD group had higher levels of the above five biomarkers, and the aMCI group had higher levels of t-Tau and Aβ_1–42_ x t-Tau. Table 2 shows the plasma levels of the 29 cytokines. Among the control, aMCI, and AD groups, the MIP1β, RANTES, and PDFG-BB levels showed a linear trend. IL1RA was significantly higher in the aMCI group than the control group, and the levels of RANTES, IL-9, and PDGF-BB were significantly lower in the AD group than the control group.

**TABLE 1:** Baseline characteristics and IMR data of the enrolled participants.^*a*^

	**Control**	**aMCI**	**AD^*b*^**	***P* value**		
**Variable**	**(*n* = 12)**	**(*n* = 51)**	**(*n* = 28)**	**P trend analysis**	**MCI vs. Control**	**AD vs. Control**
**Demographics**						
Male	3 (25.0)	11 (21.6)	7 (25.0)	na	0.532	0.663
Age, years	66.3 ± 5.9	75.6 ± 8.6	78.3 ± 8.8	na	<0.001*	<0.001*
Education, years	11.0 ± 3.9	8.2 ± 4.7	10.1 ± 5.1	na	0.026*	0.489
BMI, kg/m^2^	23.4 ± 2.1	24.7 ± 3.5	23.3 ± 3.4	na	0.064	0.708
**Cognitive test**						
Baseline MMSE	29.4 ± 0.5	24.7 ± 4.5	20.9 ± 5.4	<0.001*	<0.001*	<0.001*
CDR	0 ± 0	0.25 ± 0.34	0.77 ± 0.50	<0.001*	<0.001*	<0.001*
tCDR	0.46 ± 0.26	1.7 ± 1.7	3.4 ± 3.4	<0.001*	<0.001*	<0.001*
HVLT	22.2 ± 4.9	17.0 ± 5.7	14.6 ± 6.0	<0.001*	0.003*	<0.001*
Disease Index	11.3 ± 0.8	10.1 ± 2.1	8.2 ± 3.6	0.001*	0.01*	<0.001*
fDS	11.6 ± 1.6	9.2 ± 2.7	9.0 ± 2.7	0.006*	<0.001*	<0.001*
bDS	7.5 ± 3.0	4.3 ± 2.7	2.9 ± 2.0	<0.001*	<0.001*	<0.001*
VFT	13.9 ± 2.2	11.2 ± 4.0	8.6 ± 3.6	<0.001*	<0.001*	<0.001*
MBNT	14.5 ± 0.8	13.4 ± 1.6	13.2 ± 1.9	0.023*	0.002*	0.004*
TMTA	48.6 ± 25.6	109.5 ± 85.9	132.0 ± 95.7	0.005*	<0.001*	<0.001*
**IMR data**						
t-Tau, pg/ml	22.1 ± 3.1	25.5 ± 4.5	26.3 ± 4.5	0.006*	0.048*	0.005*
*p*-Tau181, pg/ml	3.5 ± 0.5	3.9 ± 0.7	4.1 ± 1.0	0.031*	0.168	0.026*
Aβ_1–42_, pg/ml	16.7 ± 0.7	17.1 ± 0.8	17.3 ± 1.0	0.045*	0.131	0.016*
Aβ_1–40_, pg/ml	51.8 ± 5.3	51.8 ± 4.6	51.8 ± 3.9	0.961	0.908	0.876
α-synuclein, fg/ml	108 ± 78	138 ± 80	119 ± 64	0.584	0.254	0.505
Aβ_1–42_ × t-Tau	370 ± 61	438 ± 92	458 ± 105	0.007*	0.047*	0.008*
Aβ_1–42_ × *p*-Tau181	58.1 ± 10.1	67.1 ± 13.7	71.0 ± 21.0	0.022*	0.127	0.021*
Aβ_1–42/_ Aβ_1–40_	0.32 ± 0.04	0.33 ± 0.03	0.34 ± 0.03	0.376	0.521	0.362
APOE ε4 (%)	3 (25.0)	12 (23.5)	9 (32.1)	0.521	0.797	0.651

*Aβ, amyloid β; AD, Alzheimer’s disease; aMCI, amnestic mild cognitive impairment; APOE, apolipoprotein E genotype; bDS, backward digit span; BMI, body mass index; CDR, Clinical Dementia Rating; fDS, forward digit span; HVLF, Hopkins Verbal Learning Test; IMR, ultra-sensitive immunomagnetic reduction; Modified Boston Naming Test (MBNT); MMSE, Mini-Mental Status Examination; na, not applicable; *p*-Tau181, tau phosphorylated at threonine 181; TMTA, Trail Making Test Part A; tCDR, total score of Clinical Dementia Rating; and t-Tau, total Tau. ^*a*^Data are presented as frequency (percentage) or mean ± standard deviation. ^*b*^The diagnosis of AD was made according to clinical symptoms and cognitive tests not supported by postmortem examination or *in vivo* by biomarkers. Values in bold type indicates statistical significance.*

**TABLE 2:** Plasma levels of cytokines of the enrolled participants.^*a*^

		**Control**	**aMCI**	**AD**	***P* value**		
	**Cytokine, pg/ml**	**(*n* = 12)**	**(*n* = 51)**	**(*n* = 28)**	**P trend analysis**	**MCI vs. control**	**AD vs. control**
Th1-related	IL-2	1.05 [0.91, 1.09]	1.14 [0.91, 1.56]	1.22 [0.64, 1.55]	0.447	0.165	0.258
	IFNγ	0.45 [0.42, 0.73]	0.54 [0.26, 0.70]	0.51 [0.40, 0.91]	0.943	0.861	0.627
	TNFα	5.6 [5.3, 7.0]	5.6 [4.8, 7.1]	6.1 [4.8, 8.2]		0.643	0.604
Th2-related	IL-4	0.94 [0.70, 1.14]	0.92 [0.79, 1.23]	1.05 [0.69, 1.38]	0.657	0.569	0.371
	IL-5	3.0 [1.3, 4.0]	3.2 [2.3, 4.0]	3.2 [2.3, 4.0]	0.153	0.306	0.280
	IL-6	0.30 [0.22, 0.42]	0.29 [0.20, 0.39]	0.32 [0.22, 0.49]	0.961	0.837	0.517
	IL-10	1.2 [1.0, 1.8]	0.9 [0.5, 1.5]	1.2 [0.4, 2.1]	0.979	0.438	0.925
	IL-13	0.92 [0.74, 1.31]	0.95 [0.74, 1.20]	1.00 [0.74, 1.29]	0.908	0.896	0.777
Th17-related	IL-1β	0.08 [0.07, 0.12]	0.09 [0.06, 0.13]	0.12 [0.08, 0.14]	0.536	0.699	0.168
	IL-17A	4.3 [3.1, 5.1]	4.4 [3.8, 5.1]	5.1 [3.4, 6.0]	0.195	0.540	0.149
	IL-23	5.4 [1.7, 8.9]	5.7 [3.1, 7.5]	5.2 [4.6, 8.5]	0.212	0.626	0.614
	IL-25	0.09 [0.07, 0.16]	0.06 [0.03, 0.14]	0.06 [0.04, 0.14]	0.498	0.301	0.653
	IL-31	36.1 [30.7, 43.7]	34.2 [24.1, 41.9]	34.1 [25.8, 43.5]	0.781	0.391	0.660
	sCD40L	25.5 [14.4, 34.7]	14.0 [9.3, 22.2]	12.5 [8.3, 19.7]	0.135	0.064	0.055
Chemokine	IL-8	0.90 [0.49, 1.08]	0.98 [0.66, 1.29]	1.15 [0.72, 1.33]	0.394	0.270	0.139
	IP10	569 [268, 758]	542.1 [303, 722]	379.3 [259, 568]	0.114	0.834	0.245
	MCP1	14.0 [9.0, 17.5]	11.7 [7.6, 17.1]	10.5 [6.4, 15.9]	0.190	0.558	0.362
	MIP1α	1.02 [0.82, 1.36]	1.14 [0.83, 1.33]	1.08 [0.86, 1.32]	0.698	0.739	0.615
	MIP1β	24.8 [18.1, 34.3]	18.7 [16.4, 24.3]	19.3 [16.7, 25.7]	0.027*	0.096	0.177
	RANTES	1654 [1351, 1822]	1379 [861, 1710]	1079 [812, 1537]	0.005*	0.146	0.006*
	Eotaxin	43.7 [30.7, 55.4]	38.1 [29.6, 52.7]	36.4 [28.1, 53.2]	0.302	0.753	0.379
Others	IL-1RA	49.8 [39.0, 67.3]	78.8 [62.1, 103.0]	67.9 [50.5, 85.6]	0.161	0.003*	0.118
	IL-7	7.6 [6.6, 9.3]	7.6 [6.7, 8.6]	8.2 [6.6, 10.7]	0.329	0.875	0.530
	IL-9	17.0 [16.6, 17.7]	16.4 [14.2, 18.1]	16.2 [14.6, 17.0]	0.658	0.263	0.035*
	FGF	6.7 [6.4, 7.7]	7.3 [6.4, 9.3]	7.6 [6.9, 10.1]	0.318	0.293	0.106
	GCSF	86.2 [63.5, 106.5]	92.0 [75.4, 118.2]	91.5 [68.6, 112.7]	0.920	0.381	0.638
	GM-CSF	0.18 [0.14, 0.32]	0.28 [0.17, 0.49]	0.48 [0.14, 0.79]	0.209	0.383	0.342
	PDGF-BB	373 [192, 503]	216 [116, 355]	163 [55, 276]	0.008*	0.052	0.028*
	VEGF	25.4 [17.6, 36.8]	29.1 [21.8, 43.0]	33.3 [23.0, 44.4]	0.479	0.182	0.208

AD, Alzheimer’s disease; aMCI, amnestic mild cognitive impairment; FGF, fibroblast growth factor; GCSF, granulocyte colony-stimulating factor; GM-CSF, granulocyte-macrophage colony-stimulating factor; IFNγ, interferon-gamma; IL, interleukin; IL-1RA, IL-1 receptor antagonist; IP10, IFNγ-induced protein 10; MCP1, monocyte chemoattractant protein 1; MIP1α, macrophage inflammatory protein 1-alpha; PDGF-BB, platelet-derived growth factor-BB; RANTES, regulated upon activation, normal T cell expressed and secreted; sCD40L, soluble CD40 ligand; Th, T helper; TNFα, tumor necrosis factor-alpha; VEGF, vascular endothelial growth factor. ^*a*^Data are presented as median [25th, 75th percentile]; *P < 0.05. Values in bold type indicates statistical significance.

### Association Between Plasma Biomarkers and Cognitive Test Results

We examined whether the plasma biomarkers were associated with cognitive test results. As shown in the supplementary data (Supplementary Tables 1–3). Several plasma biomarkers were associated with cognitive test results in the three groups. In control group, the levels of t-Tau/Aβ_1–42_ ratio, IL-4, IL-5, IL-7, IL-9, IL-13, IL-17A, eotaxin, FGF, MIP1β, PDFG-BB, RANTES, IL-23, IL-25, and sCD40L were associated with the cognitive test results. In the aMCI group, the levels of Aβ_1–40_, Aβ_1–42_/ Aβ_1–40_ ratio, *p*-Tau/t-Tau ratio, IL-1β, IL-4, IL-8, IL-10, FGF, INFγ, IP-10, MIP1β, PDFG-BB, TNFα, IL-23, IL-31, and sCD40L were associated with the cognitive test results. In the AD group, the levels of t-Tau, Aβ_1–40_, Aβ_1–42_, α-Syn, t-Tau/Aβ_1–42_ ratio, Aβ_1–42_/ Aβ_1–40_ ratio, IL-1β, IL-2, IL-5, IL-10, FGF, GM-CSF, INFγ, MCP1, MIP1α, PDGF-BB, RANTES, IL-25, and IL-31 were associated with the cognitive test results.

### Association Between Plasma Biomarkers and Annual Change in MMSE Score

We then examined the association between plasma biomarkers and annual changes in the MMSE score in the aMCI and AD groups. For both groups, there was no significant association of the Aβ and tau biomarkers with the annual change in MMSE score (Table 3), except for Aβ_1–40_ in patients with AD (*r* = −0.684, *p* = 0.042). However, several cytokine levels were associated with annual MMSE changes. The aMCI group showed that higher baseline levels of IL-2 (*r* = 0.420, *p* = 0.041), sCD40L (*r* = 0.419, *p* = 0.041), IL-8 (*r* = 0.410, *p* = 0.047), and VEGF (*r* = 0.491, *p* = 0.017) were significantly correlated with a lower cognitive decline. In contrast, the AD group showed that the higher baseline levels of IFNγ (*r* = −0.701, *p* = 0.036), IL-5 (*r* = −0.756, *p* = 0.019), IL-17A (*r* = −0.759, *p* = 0.021), IL-25 (*r* = −0.840, *p* = 0.036), and FGF (*r* = −0.780, *p* = 0.013) were significantly correlated with more rapid cognitive decline. Interestingly, the MCI group had positive **associations** of cytokine levels with annual MMSE changes, while the AD group had negative associations of cytokine levels and annual MMSE changes.

**TABLE 3:** Association of IMR data and cytokine levels with annual change in MMSE score.^*a*^

	**Variable**	**aMCI**		**AD**	
		**Partial correlation**	***P* value**	**Partial correlation**	***P* value**
IMR	t-Tau,	−0.067	0.757	−0.070	0.859
	Aβ_1–42_	−0.039	0.858	0.024	0.952
	*p*-Tau181	−0.173	0.419	0.168	0.666
	Aβ_1–40_	−0.173	0.409	−**0.684**	0.042*
	α-synuclein	0.234	0.271	0.232	0.548
	Aβ_1–42_ × t-Tau	−0.073	0.735	−0.109	0.781
	Aβ_1–42_ × Aβ_1–40_	0.127	0.554	0.419	0.261
	*p*-Tau × t-Tau	−0.167	0.435	0.364	0.336
Th1-related	IL-2	**0.420**	0.041*	−0.025	0.949
	IFNγ	0.340	0.122	−**0.701**	0.036*
	TNFα	0.185	0.387	0.194	0.617
Th2-related	IL-4	0.350	0.094	0.487	0.183
	IL-5	0.196	0.358	−**0.756**	0.019*
	IL-6	0.205	0.349	0.112	0.811
	IL-10	−0.065	0.792	0.600	0.155
	IL-13	0.002	0.991	0.053	0.893
Th17-related	IL-1β	0.280	0.186	−0.266	0.524
	IL-17A	0.297	0.158	−**0.745**	0.021*
	IL-23	−0.069	0.760	−0.492	0.178
	IL-25	−0.158	0.519	−**0.840**	0.036*
	IL-31	0.264	0.213	−0.159	0.684
	sCD40L	**0.419**	0.041*	−0.074	0.849
Chemokine	IL-8	**0.410**	0.047*	0.460	0.213
	IP10	0.212	0.319	0.041	0.917
	MCP1	0.059	0.784	0.600	0.087
	MIP1α	0.292	0.167	−0.348	0.358
	MIP1β	−0.055	0.798	0.356	0.348
	RANTES	0.134	0.532	0.233	0.547
	Eotaxin	0.316	0.133	0.444	0.231
Others	IL-1RA	0.229	0.281	−0.201	0.603
	IL-7	0.172	0.423	0.008	0.984
	IL-9	0.111	0.606	0.098	0.801
	FGF	0.104	0.630	−**0.780**	0.013*
	GCSF	0.321	0.126	0.261	0.497
	GM-CSF	0.386	0.139	−0.996	0.055
	PDGF-BB	0.147	0.494	0.011	0.977
	VEGF	**0.491**	0.017*	−0.655	0.056

AD, Alzheimer’s disease; aMCI; amnestic mild cognitive impairment; FGF, fibroblast growth factor; GCSF, granulocyte colony-stimulating factor; GM-CSF, granulocyte-macrophage colony-stimulating factor; IFNγ, interferon-gamma; IL, interleukin; IL-1RA, IL-1 receptor antagonist; IP10, IFNγ-induced protein 10; MCP1, monocyte chemoattractant protein 1; MIP1α, macrophage inflammatory protein 1-alpha; MMSE, Mini Mental Status Examination; PDGF-BB, platelet-derived growth factor-BB; RANTES, regulated upon activation, normal T cell expressed and secreted; sCD40L, soluble CD40 ligand; Th, T helper; TNFα, tumor necrosis factor-alpha; and VEGF, vascular endothelial growth factor. ^*a*^Adjusted for age, education level, body mass index, and apolipoprotein E containing ε4. *P < 0.05. Values in bold type indicates statistical significance.

### Discriminatory Capacity of Plasma Biomarkers for Identifying Cognitive Decline

Several cytokines were correlated with annual MMSE score changes in the partial correlation analysis. We then compared these cytokines with the Aβ and tau biomarkers to assess their potential discriminatory capacity for identifying cognitive decline (a change in the annual MMSE score ≥ 2) in the aMCI (event number = 4) and the AD (event number = 4) groups. As shown in Table 4, none of the Aβ and tau biomarkers were able to predict cognitive decline in the aMCI and AD groups. In terms of cytokine levels, IL-2 had a satisfactory discriminatory capacity for detecting cognitive decline in the aMCI group (AUC = 85.7%, 95% CI = 69.7–100%). IL-25 had the best discriminatory capacity for identifying cognitive decline in the AD group; however, it did not reach statistical significance. The optimal cutoff was ≤ 1 pg/ml, with a sensitivity of 100% (95% CI = 39.8–100%) and specificity of 67.9% (95% CI = 47.6–84.1%). When assessing IL-2 with Aβ and tau biomarkers in the aMCI group, the combined AUC values increased to 88.4%. In contrast, when assessing IL-25 with each IMR biomarker in the AD group, all the combined AUC values were increased, but none of them were significant (Table 4).

**TABLE 4:** Diagnostic utility of IMR data and cytokine levels for identifying cognitive decline (annual decline in MMSE score ≥ 2) in the aMCI and AD groups.

	**Parameter**	**AUC, % (95% CI)**	
		**aMCI**	**AD**
IMR data	t-Tau,	56.3 (22.4–90.1)	52.8 (16.8–88.7)
	Aβ_1–42_	66.1 (28.4–100.0)	55.6 (12.9–98.2)
	*p*-Tau181	66.1 (42.3–89.9)	55.6 (14.8–96.3)
	Aβ_1–40_	64.3 (26.3–100.0)	52.8 (10.0–84.4)
	α-synuclein	66.1 (42.9–89.2)	52.8 (5.7–88.7)
	Aβ_1–42_ × t-Tau	46.4 (19.5–73.4)	52.8 (11.3–83.2)
	Aβ_1–42_ × Aβ_1–40_	69.6 (42.2–97.1)	50.0 (9.8–90.2)
	*p*-Tau × t-Tau	58.0 (33.1–83.0)	52.8 (11.1–94.5)
Cytokine	IL-2	85.7 (69.7–100.0)*^a^	50.0 (2.9–97.1)
	IFNγ	43.8 (23.0–64.5)	29.2 (0.0–76.4)
	IL-5	71.9 (38.5–100.0)	41.7 (0.0–91.3)
	IL-17A	67.9 (42.5–93.2)	44.4 (0.0–91.9)
	IL-25	53.6 (16.9–90.3)	73.6 (44.3–100.0)
	sCD40L	67.9 (34.9–100.0)	66.7 (33.7–99.6)
	FGF	62.5 (33.9–91.1)	66.7 (28.6–100.0)
	IL-8	69.1 (33.7–100.0)	73.6 (31.3–100.0)
	VEGF	67.4 (41.1–93.7)	43.1 (0.0–86.9)
Combined		IL-2 + IMR data	IL-25 + IMR data
	t-Tau	88.4 (74.2–100.0)*	69.4 (37.5–100.0)
	Aβ_1–42_	85.7 (69.7–100.0)*	72.2 (33.9–100.0)
	*p*-Tau181	84.8 (68.6–100.0)*	61.1 (18.4–100.0)
	Aβ_1–40_	86.6 (69.4–100.0)*	75.0 (46.5–100.0)
	α-synuclein	88.4 (76.1–100.0)*	63.9 (19.3–100.0)
	Aβ_1–42_ × t-Tau	88.4 (75.3–100.0)*	72.2 (41.7–100.0)
	Aβ_1–42_ × Aβ_1–40_	87.5 (71.8–100.0)*	72.2 (38.7–100.0)
	*p*-Tau × t-Tau	87.5 (72.5–100.0)*	55.6 (4.1–100.0)

Aβ, amyloid β; AD, Alzheimer’s disease; aMCI, amnestic mild cognitive impairment; AUC, area under the curve; FGF, fibroblast growth factor; CI, confidence interval; GM-CSF, granulocyte-macrophage colony-stimulating factor; IFNγ; interferon-gamma; IL, interleukin; IMR, ultra-sensitive immunomagnetic reduction; MMSE, Mini-Mental Status Examination; *p*-Tau181, tau phosphorylated at threonine 181; sCD40L, soluble CD40 ligand; Th helper; t-Tau, total Tau; and VEGF, vascular endothelial growth factor. ^*a*^The optimal cutoff was ≤ 1 pg/ml with a sensitivity of 100% (39.8–100%) and specificity of 67.9% (47.6–84.1%). *P < 0.05. Values in bold type indicates statistical significance.

### Temporal Association of IL-2, Aβ, and Tau Between Controls, aMCI, and AD

**FIGURE 1:**
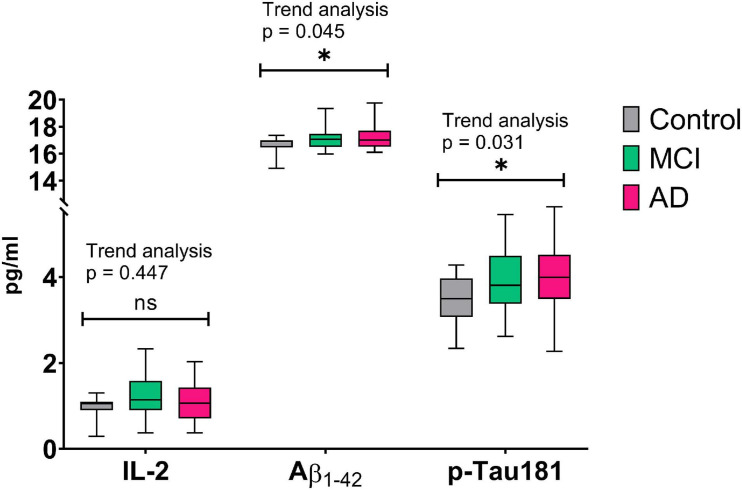
Temporal association of plasma biomarkers between controls, aMCI, and AD groups. Aβ, amyloid β; AD, Alzheimer’s disease; aMCI, amnestic mild cognitive impairment; IL, interleukin; ns, not statistically significant. *Indicates statistical significance.

Finally, we examined the temporal association in plasma IL-2, Aβ_1–42_, and *p*-Tau181 levels between the three groups (Figure 1). We found that there was a linear trend for plasma Aβ_1–42_ (*p* = 0.045) and *p*-Tau181 (*p* = 0.031) levels; however, the linear trend was not observed for plasma IL-2 levels. Indeed, the aMCI group had higher IL-2 levels (1.19 ± 0.53) than the control (0.95 ± 0.29) and AD (1.09 ± 0.62) groups, but there was no statistical significance (aMCI vs controls, *p* = 0.165; aMCI vs AD, *p* = 0.460).

## Discussion

This is the first clinical study to compare the discriminatory capacity of plasma cytokines versus plasma Aβ and tau biomarkers for identifying cognitive decline in patients with aMCI or AD. We found that plasma Aβ and tau biomarkers were not associated with annual changes in the MMSE score in both the aMCI and AD groups, while several plasma cytokines were associated with cognitive test results and annual changes in the MMSE score. In the aMCI group, higher baseline plasma levels of IL-2, sCD40L, and VEGF were associated with a lower cognitive decline, and in the AD group, higher baseline plasma levels of IFNγ, IL-5, IL-17A, IL-25, FGF, GM-CSF, and VEGF were associated with a more rapid cognitive decline. Among these plasma cytokines, plasma levels of IL-2 showed satisfactory discriminatory potential to identify cognitive decline in the aMCI group, and the AUC values were slightly increased when combining plasma levels of IL-2 with plasma levels of Aβ and tau biomarkers; however, in the AD group, all the investigated plasma cytokines did not reach significance for identifying cognitive decline. Briefly, our study suggests that initial plasma levels of several cytokines were associated with subsequent annual MMSE changes in patients with aMCI or AD, and that plasma levels of IL-2 may be a potential biomarker to detect rapid cognitive decline in the aMCI group.

In addition to Aβ plaques and neurofibrillary tangles, neuroinflammation in the central nervous system (CNS) has been suggested to play a key role in the pathology of AD (Heneka et al., 2015; Heppner et al., 2015). The CNS-intrinsic factors or systemic influences can induce neuroinflammatory responses in the brain, and activation of microglia and astroglia triggers an innate immune response characterized by the release of inflammatory mediators, including cytokines (Heneka et al., 2015; Heppner et al., 2015). This neuroinflammation contributes to disease progression and severity, and sufficiently drives the AD pathology. Our study found that higher baseline plasma levels of IL-2 were associated with a lower cognitive decline, and that plasma levels of IL-2 had a satisfactory discriminatory ability to detect cognitive decline in the aMCI group. Evidence suggests that IL-2 is involved in CNS development, normal brain physiology, and homeostatic repair mechanisms (Petitto et al., 2012). IL-2-knockout mice display impaired learning and memory reminiscent of AD as well as cytoarchitectural modifications and a reduced concentration of brain-derived neurotropic factor in the hippocampus (Petitto et al., 1999, 2012; Beck et al., 2005). An animal study found that peripheral low-dose IL-2 treatment could restore cognition in a mouse model of AD (Dansokho et al., 2016). Another animal study reported that low-dose IL-2 treatment improved the amyloid pathology, synaptic failure, and memory function in AD mice (Alves et al., 2017). The beneficial effects of IL-2 may be associated with amplification of regulatory T cells without a significant impact on conventional effector T cells and other immune effectors (Dansokho et al., 2016; Alves et al., 2017). In our study, patients with aMCI who had higher plasma levels of IL-2 may have more neuroprotective effects than those with lower plasma levels of IL-2, and these patients may thus have lesser cognitive decline.

In our study, the plasma levels of Aβ and tau biomarkers did not correlate with cognitive decline. Evidence suggests that the Aβ accumulation and tau-mediated neuronal injury and dysfunction precede clinical symptoms of aMCI and AD, and that substantial Aβ-plaque deposition alone may have no immediate effects on cognitive function (Petersen et al., 2009; Jack et al., 2013). The Aβ biomarkers plateau in the stage of aMCI and AD; therefore, cognitive decline does not significantly correlate with the rate of Aβ_1–42_ accumulation in the CSF. Moreover, this demanding process is mediated by individual differences in brain resiliency, cognitive reserve, and comorbid pathological brain changes (Jack et al., 2013). The heterogeneous clinical presentation and pathological patterns of AD might also explain the non-linear relationship of tau biomarkers with cognitive impairment at the stage of aMCI and AD (Tatsuoka et al., 2013). For example, a recent neuroimaging study reported that patients with AD could be clustered into three subtypes with distinct topographical features of cortical atrophy and tau deposition (Jeon et al., 2019). Another study used multiple biomarkers (e.g., hippocampal volume, PET amyloid deposition, and CSF tau protein) and suggest that the neurobiological processes both act independently and interact in a nonlinear fashion during progression from aMCI to AD (Popescu et al., 2020).

For the control, aMC, and AD groups, we find a linear trend for plasma levels of Aβ_1–42_ and *p*-Tau181; however, the linear trend was not observed for plasma levels of IL-2. The aMCI group had higher plasma levels of IL-2 than those of the control and AD groups, while they were not statistically significant. The mechanisms for the above study findings might be related to the beneficial role of IL-2 for AD pathology (Dansokho et al., 2016; Alves et al., 2017). In the aMCI group, higher plasma levels of IL-2 were associated with a lower cognitive decline. Therefore, the aMCI group with lower plasma levels of IL-2 might have higher risk of developing AD than those with higher plasma levels of IL-2. This might explain why the AD group might have lower plasma levels of IL-2 than the aMCI group. This finding warrants future large-scale studies into the association between IL-2 levels and AD conversion.

For Aβ measurement, our study used IMR assay which is quite different from the immunoprecipitation-mass spectrometry (IP-MS) and the single-molecule array (SIMOA) assays. The IMR assay quantifies the concentrations of analytes in a sample by measuring the percentage magnetic signal reduction after immunocomplex formation at the surface of magnetic nanobeads, and the magnetic signals are detected by a powerful magnetic sensor called a superconducting quantum interference device (Lue et al., 2019). The IP-MS assay quantifies Aβ-related peptides of different mass in mass spectrometry after they have been isolated and enriched from abundant plasma proteins by immunoprecipitation using the specific affinity of an antibody (Nakamura et al., 2018). The SIMOA assay is based on the use of capture antibodies coupled to paramagnetic beads and biotinylated detection antibodies, which in turn are detected by the action of a reporter enzyme: streptavidin β-galactosidase and resorufin β-D-galactopyranoside as the fluorescence substrate (Lue et al., 2019). For *p*-Tau181, the IMR assay differs from the SIMOA assay (Karikari et al., 2020, 2021) and the Meso Scale Discovery platform (Mielke et al., 2018). Importantly, the lower limit of quantification varies across different analytic methods. For example, in the current study using IMR assay, the lower limit of quantification of *p*-Tau181 was 0.0196 pg/ml; however, the lower limit of quantification of *p*-Tau181 was 0.5 pg/ml in SIMOA assay (Karikari et al., 2020). Therefore, our results may be affected by analytic differences and need to be validated for cross-method comparisons.

Our study has limitations that need to be considered when interpreting the results. First, the NIA-AA research framework defines AD as a biological construct rather than a clinical syndrome, and the diagnosis of AD focuses on Aβ deposition, pathologic tau, and neurodegeneration (Jack et al., 2018). Our study population was limited to patients who were clinically assessed and had positive findings on cognitive tests. We could not obtain CSF biomarkers and amyloid or tau positron emission tomography scans to define and stage the disease cross its entire spectrum as suggested by NIA-AA 2018 (Jack et al., 2018). Second, because of the absence of a transporter to the brain, IL-2 access into the brain is limited (Banks et al., 2004). Therefore, the correlation between peripheral levels of IL-2 and annual cognitive change may not be generalizable to the correlation between central levels of IL-2 and annual cognitive change. Third, cognitive decline is closely coupled with neurodegenerative biomarkers, such as hippocampal atrophy (Jack et al., 2013). The association between IL-2 and neurodegenerative biomarkers remains to be determined. Fourth, since the participants were recruited at a memory clinic, the findings of our study may not be generalizable to other study populations, such as community-dwelling elderly people. Fifth, our sample size was small. The low sample size in the control and ADD groups could bias the analysis because of overfitting due to low number of events per variable in the model therefore. Besides, the statistical power may also be decreased.

This is the first study to compare the discriminatory ability of identifying cognitive decline between cytokines, Aβ, and tau biomarkers in patients with aMCI and AD. We found that Aβ and tau biomarkers did not significantly predict annual cognitive change in both patients with aMCI and AD. Several cytokines were significantly associated with cognitive test results and annual cognitive change in patients with aMCI and AD. Plasma IL-2 levels had a satisfactory discriminating ability to detect cognitive decline in the aMCI group, and the discriminating potential was slightly increased when combined with Aβ and tau biomarkers. However, different inflammatory mechanisms might be responsible for different phases of neuroinflammation in aMCI and AD. Our study findings need to be replicated in future large longitudinal studies.

## Data Availability Statement

The raw data supporting the conclusions of this article will be made available by the authors, without undue reservation.

## Ethics Statement

The studies involving human participants were reviewed and approved by the Institutional Review Board for the Protection of Human Subjects at the Tri-Service General Hospital (TSGHIRB 1-107-05-111). Written informed consent was obtained from all participants in the study.

## Author Contributions

C-SL managed the literature review, conducted the statistical analyses, interpreted the results, and wrote the first draft of the manuscript. C-LT played a major role in the acquisition of data and revised the manuscript for intellectual content. G-YL, C-SC, T-CY, H-TC, and M-WS interpreted the data and revised the manuscript for intellectual content. J-TL designed the study and provided conceptualization and theory used to integrate the findings, and edited the manuscript. Y-KL and C-KT interpreted the results and provided feedback and comments on the various versions of the manuscript. Y-FS provided the conceptualization and theory used to integrate the findings and edited the manuscript. F-CY designed the study, directed the data collection, provided the overall scientific supervision, interpreted the results, and edited the manuscript. All authors read and approved the final manuscript.

## Conflict of Interest

The authors declare that the research was conducted in the absence of any commercial or financial relationships that could be construed as a potential conflict of interest.
