# Supplementary material for: Better Identification of Cognitive Decline With Interleukin-2 Than With Amyloid and Tau Protein Biomarkers in Amnestic Mild Cognitive Impairment
Source: Front Aging Neurosci. 2021 May 28;13:670115. doi: 10.3389/fnagi.2021.670115 (PMC8193360; doi:10.3389/fnagi.2021.670115)
Supplement: Supplementary file 1 [file Table_1.DOCX]

**eTable 1.** The relationship between IMR/Cytokine and cognitive test in the control group.

| IMR/Cytokine | Baseline MMSE | tCDR | HVLT | Disease Index | fDS | bDS | VFT | MBNT | TMTA |
| --- | --- | --- | --- | --- | --- | --- | --- | --- | --- |
| IMR data |  |  |  |  |  |  |  |  |  |
| t-Tau | -0.17 | 0.20 | -0.27 | -0.29 | -0.18 | -0.19 | 0.19 | -0.58* | 0.48 |
| Aβ_1-42_ | 0.22 | 0.31 | -0.14 | -0.05 | -0.29 | -0.08 | 0.12 | -0.39 | 0.35 |
| p-Tau181 | -0.02 | -0.07 | -0.21 | -0.29 | -0.36 | -0.24 | 0.52 | -0.13 | 0.30 |
| Aβ_1-4_0 | -0.27 | 0.46 | -0.02 | -0.24 | -0.37 | -0.42 | -0.33 | 0.00 | 0.37 |
| α-synuclein | -0.02 | -0.27 | 0.18 | 0.28 | 0.32 | 0.38 | 0.27 | -0.22 | -0.40 |
| Aβ_1-42_ × t-Tau | -0.22 | 0.15 | -0.37 | -0.40 | -0.16 | -0.23 | 0.20 | -0.63* | 0.53 |
| Aβ_1-42_ × Aβ_1-40_ | 0.22 | -0.31 | 0.04 | 0.33 | 0.27 | 0.37 | 0.34 | -0.09 | -0.31 |
| p-Tau × t-Tau | 0.02 | -0.11 | 0.05 | 0.03 | -0.23 | -0.11 | 0.27 | 0.30 | -0.08 |
| Th1 cell-related |  |  |  |  |  |  |  |  |  |
| IL-2 | 0.22 | -0.09 | -0.06 | -0.52 | 0.01 | 0.17 | -0.13 | -0.18 | 0.13 |
| IFNγ | 0.66 | -0.47 | 0.09 | -0.46 | 0.25 | 0.44 | -0.04 | 0.19 | -0.38 |
| TNFα | 0.34 | 0.03 | -0.12 | -0.21 | -0.10 | 0.15 | -0.18 | -0.29 | 0.26 |
| Th2 cell-related |  |  |  |  |  |  |  |  |  |
| IL-4 | 0.12 | -0.36 | 0.34 | -0.58* | 0.53 | 0.56 | 0.15 | 0.14 | -0.15 |
| IL-5 | 0.35 | -0.57 | 0.40 | -0.06 | 0.55 | 0.74* | 0.38 | -0.05 | -0.51 |
| IL-6 | 0.43 | -0.30 | -0.06 | -0.37 | 0.32 | 0.52 | 0.43 | -0.34 | -0.01 |
| IL-10 | 0.00 | -0.28 | -0.14 | -0.66 | 0.28 | 0.01 | 0.13 | 0.36 | -0.13 |
| IL-13 | 0.10 | -0.39 | 0.16 | -0.61* | 0.59* | 0.52 | 0.34 | 0.06 | -0.16 |
| Th17 cell-related |  |  |  |  |  |  |  |  |  |
| IL-1β | 0.57 | -0.35 | -0.07 | -0.22 | 0.33 | 0.53 | 0.31 | -0.23 | -0.18 |
| IL-17A | 0.37 | -0.31 | 0.52 | -0.38 | 0.51 | 0.67* | 0.15 | 0.30 | -0.32 |
| IL-23 | 0.00 | -0.33 | 0.80* | 0.22 | 0.35 | 0.38 | 0.18 | 0.70 | -0.44 |
| IL-25 | 0.00 | -0.28 | 0.12 | 0.61 | 0.48 | 0.57 | -0.13 | 0.33 | -0.78* |
| IL-31 | 0.32 | -0.56 | -0.13 | -0.14 | 0.37 | 0.45 | 0.29 | -0.01 | -0.52 |
| sCD40L |  |  |  |  |  |  |  |  |  |
| CXC chemokine | -0.25 | 0.33 | -0.18 | -0.05 | 0.20 | 0.05 | -0.38 | -0.35 | 0.03 |
| IL-8 | 0.47 | 0.21 | -0.45 | 0.01 | 0.14 | 0.26 | -0.35 | -0.43 | -0.11 |
| IP10 |  |  |  |  |  |  |  |  |  |
| CC chemokine | 0.27 | 0.35 | -0.03 | -0.30 | -0.04 | 0.13 | -0.40 | -0.20 | 0.34 |
| MCP1 | 0.27 | -0.53 | 0.20 | -0.45 | 0.24 | 0.36 | 0.01 | 0.03 | -0.08 |
| MIP1α | 0.61* | -0.66* | 0.21 | -0.34 | 0.54 | 0.72* | 0.16 | 0.21 | -0.50 |
| MIP1β | 0.22 | 0.15 | 0.03 | -0.05 | -0.27 | -0.06 | -0.65* | 0.03 | 0.09 |
| RANTES | 0.32 | -0.21 | 0.42 | -0.66* | 0.55 | 0.55 | -0.09 | 0.37 | -0.15 |
| Eotaxin |  |  |  |  |  |  |  |  |  |
| Others | 0.35 | 0.01 | 0.19 | -0.25 | 0.52 | 0.52 | -0.10 | 0.02 | -0.21 |
| IL-1RA | 0.27 | -0.43 | 0.39 | -0.49 | 0.64* | 0.65* | 0.24 | 0.36 | -0.38 |
| IL-7 | 0.71* | 0.12 | -0.01 | 0.16 | 0.06 | 0.36 | -0.60* | 0.04 | -0.25 |
| IL-9 | 0.17 | -0.65* | 0.36 | 0.05 | 0.36 | 0.52 | 0.66* | 0.00 | -0.37 |
| FGF | 0.22 | -0.27 | 0.42 | -0.40 | 0.40 | 0.54 | 0.00 | 0.26 | -0.17 |
| GCSF | 0.29 | 0.45 | -0.15 | -0.67 | -0.15 | 0.15 | -0.58 | -0.29 | 0.00 |
| GM-CSF | 0.61* | -0.61* | 0.12 | -0.26 | 0.37 | 0.66* | 0.31 | 0.01 | -0.48 |
| PDGF-BB | 0.07 | -0.04 | 0.37 | -0.09 | 0.02 | 0.23 | -0.24 | 0.07 | -0.14 |
| VEGF | 0.81* | -0.26 | -0.03 | -0.20 | 0.34 | 0.64* | -0.04 | 0.12 | -0.56 |

^Abbreviations: FGF, fibroblast growth factor; GCSF, granulocyte colony-stimulating factor; GM-CSF, granulocyte-macrophage colony-stimulating factor; IFNγ; interferon-gamma; IL, interleukin; IL-1RA, IL-1 receptor antagonist; IP10, IFNγ-induced protein 10; MCI, mild cognitive impairment; MCP1; monocyte chemoattractant protein 1; MIP1α, macrophage inflammatory protein 1-alpha; PDGF-BB, platelet-derived growth factor-BB; RANTES, regulated upon activation, normal T cell expressed and secreted; sCD40L, soluble CD40 ligand; Th, T helper; TNFα, tumor necrosis factor-alpha; and VEGF, vascular endothelial growth factor;^

* *P* value <0.05.

**eTable 2.** The relationship between IMR/Cytokine and cognitive test in the MCI group.

| IMR/Cytokine | Baseline MMSE | tCDR | HVLT | Disease Index | fDS | bDS | VFT | MBNT | TMTA |
| --- | --- | --- | --- | --- | --- | --- | --- | --- | --- |
| IMR data |  |  |  |  |  |  |  |  |  |
| t-Tau | -0.23 | 0.14 | -0.24 | -0.26 | -0.12 | -0.10 | -0.05 | 0.26 | 0.09 |
| Aβ_1-42_ | -0.09 | 0.01 | -0.07 | -0.20 | -0.03 | 0.03 | 0.04 | 0.21 | -0.09 |
| p-Tau181 | -0.16 | 0.21 | -0.16 | -0.07 | 0.02 | 0.01 | 0.15 | 0.20 | 0.03 |
| Aβ_1-4_0 | -0.38* | 0.16 | -0.23 | -0.29* | 0.00 | -0.39* | 0.09 | 0.12 | 0.18 |
| α-synuclein | 0.01 | -0.21 | -0.06 | 0.02 | -0.04 | -0.06 | 0.00 | 0.02 | 0.04 |
| Aβ_1-42_ × t-Tau | -0.23 | 0.15 | -0.25 | -0.26 | -0.11 | -0.11 | -0.08 | 0.21 | 0.13 |
| Aβ_1-42_ × Aβ_1-40_ | 0.27 | -0.16 | 0.11 | 0.17 | -0.01 | 0.32* | -0.06 | 0.01 | -0.17 |
| p-Tau × t-Tau | 0.15 | 0.07 | 0.13 | 0.21 | 0.13 | 0.10 | 0.28* | -0.04 | -0.09 |
| Th1 cell-related |  |  |  |  |  |  |  |  |  |
| IL-2 | -0.24 | 0.07 | -0.13 | -0.15 | -0.11 | -0.06 | -0.03 | 0.05 | 0.12 |
| IFNγ | -0.40* | 0.06 | -0.39* | -0.19 | -0.20 | -0.22 | -0.06 | 0.09 | 0.35* |
| TNFα | -0.27 | -0.08 | -0.28* | -0.17 | -0.09 | -0.10 | 0.10 | -0.01 | 0.13 |
| Th2 cell-related |  |  |  |  |  |  |  |  |  |
| IL-4 | -0.36* | 0.14 | -0.26 | -0.08 | -0.22 | -0.25 | -0.07 | -0.02 | 0.21 |
| IL-5 | -0.16 | 0.07 | -0.02 | -0.16 | -0.07 | -0.10 | 0.02 | 0.07 | 0.07 |
| IL-6 | -0.15 | -0.10 | 0.05 | -0.06 | 0.01 | 0.04 | 0.18 | 0.20 | -0.18 |
| IL-10 | -0.45* | 0.10 | -0.48* | -0.44* | -0.31 | -0.24 | 0.00 | 0.06 | 0.39* |
| IL-13 | -0.21 | 0.05 | -0.25 | -0.03 | -0.08 | -0.20 | -0.07 | 0.12 | 0.07 |
| Th17 cell-related |  |  |  |  |  |  |  |  |  |
| IL-1β | -0.13 | -0.02 | -0.30* | 0.03 | -0.17 | 0.03 | -0.07 | 0.27 | 0.00 |
| IL-17A | -0.22 | 0.10 | -0.21 | -0.04 | -0.11 | -0.19 | 0.02 | -0.08 | 0.17 |
| IL-23 | 0.21 | 0.10 | 0.20 | 0.16 | 0.32* | 0.25 | 0.01 | 0.09 | -0.20 |
| IL-25 | -0.15 | 0.14 | -0.15 | 0.07 | 0.08 | 0.12 | -0.10 | 0.09 | 0.05 |
| IL-31 | 0.13 | 0.01 | 0.10 | 0.31* | 0.05 | 0.13 | 0.05 | 0.03 | -0.01 |
| sCD40L |  |  |  |  |  |  |  |  |  |
| CXC chemokine | -0.36* | 0.10 | -0.30* | -0.18 | -0.26 | -0.35* | -0.10 | 0.05 | 0.39* |
| IL-8 | -0.17 | -0.06 | -0.07 | -0.50* | -0.05 | -0.09 | 0.14 | -0.05 | 0.12 |
| IP10 |  |  |  |  |  |  |  |  |  |
| CC chemokine | -0.19 | -0.04 | -0.07 | -0.25 | -0.11 | -0.02 | 0.05 | 0.06 | -0.01 |
| MCP1 | -0.14 | -0.20 | -0.09 | -0.25 | -0.10 | -0.13 | 0.10 | -0.20 | 0.08 |
| MIP1α | 0.11 | -0.40* | 0.17 | 0.10 | 0.34* | 0.23 | 0.30* | 0.12 | -0.16 |
| MIP1β | -0.03 | -0.25 | 0.03 | -0.22 | 0.02 | 0.18 | 0.26 | -0.02 | 0.08 |
| RANTES | -0.23 | -0.03 | -0.24 | -0.21 | -0.11 | -0.09 | 0.01 | -0.07 | 0.21 |
| Eotaxin |  |  |  |  |  |  |  |  |  |
| Others | -0.17 | -0.16 | -0.22 | -0.20 | -0.25 | -0.23 | 0.02 | -0.18 | 0.14 |
| IL-1RA | -0.06 | -0.03 | -0.15 | 0.06 | -0.10 | -0.09 | 0.04 | -0.12 | 0.18 |
| IL-7 | -0.09 | -0.16 | -0.02 | -0.07 | -0.08 | 0.06 | 0.08 | 0.07 | 0.10 |
| IL-9 | -0.08 | -0.12 | -0.17 | 0.17 | 0.00 | 0.08 | 0.14 | 0.28* | -0.14 |
| FGF | -0.17 | -0.04 | -0.19 | -0.16 | -0.09 | -0.12 | -0.03 | -0.05 | 0.09 |
| GCSF | 0.07 | -0.35 | 0.20 | 0.13 | 0.06 | 0.36 | 0.14 | 0.31 | -0.01 |
| GM-CSF | 0.11 | -0.31* | 0.10 | 0.05 | 0.07 | 0.40* | 0.37* | 0.20 | -0.15 |
| PDGF-BB | -0.13 | -0.06 | -0.17 | -0.18 | -0.14 | -0.06 | -0.19 | 0.02 | 0.04 |
| VEGF | 0.14 | -0.36* | 0.03 | 0.11 | 0.04 | 0.27 | 0.26 | 0.01 | 0.05 |

^Abbreviations: FGF, fibroblast growth factor; GCSF, granulocyte colony-stimulating factor; GM-CSF, granulocyte-macrophage colony-stimulating factor; IFNγ; interferon-gamma; IL, interleukin; IL-1RA, IL-1 receptor antagonist; IP10, IFNγ-induced protein 10; MCI, mild cognitive impairment; MCP1; monocyte chemoattractant protein 1; MIP1α, macrophage inflammatory protein 1-alpha; PDGF-BB, platelet-derived growth factor-BB; RANTES, regulated upon activation, normal T cell expressed and secreted; sCD40L, soluble CD40 ligand; Th, T helper; TNFα, tumor necrosis factor-alpha; and VEGF, vascular endothelial growth factor;^

* *P* value <0.05.

**eTable 3.** The relationship between IMR/Cytokine and cognitive test in the AD group^#^.

| IMR/Cytokine | Baseline MMSE | tCDR | HVLT | Disease Index | fDS | bDS | VFT | MBNT | TMTA |
| --- | --- | --- | --- | --- | --- | --- | --- | --- | --- |
| IMR data |  |  |  |  |  |  |  |  |  |
| t-Tau | -0.37* | -0.09 | -0.11 | -0.01 | -0.27 | -0.27 | 0.00 | -0.04 | 0.06 |
| Aβ_1-42_ | -0.19 | -0.18 | -0.30 | -0.01 | -0.42* | -0.27 | 0.03 | 0.14 | -0.15 |
| p-Tau181 | -0.29 | -0.14 | -0.01 | -0.04 | -0.20 | -0.26 | 0.04 | 0.19 | -0.03 |
| Aβ_1-4_0 | 0.29 | 0.07 | 0.02 | 0.01 | 0.40* | 0.23 | 0.07 | -0.03 | 0.03 |
| α-synuclein | 0.25 | -0.13 | 0.22 | 0.29 | 0.31 | 0.24 | 0.25 | 0.50* | -0.36 |
| Aβ_1-42_ × t-Tau | -0.49* | -0.03 | -0.09 | -0.05 | -0.29 | -0.31 | 0.01 | -0.14 | 0.18 |
| Aβ_1-42_ × Aβ_1-40_ | -0.35 | -0.17 | -0.20 | -0.04 | -0.49* | -0.27 | -0.02 | 0.15 | -0.11 |
| p-Tau × t-Tau | -0.13 | -0.16 | 0.23 | 0.04 | 0.09 | -0.11 | 0.01 | 0.23 | 0.00 |
| Th1 cell-related |  |  |  |  |  |  |  |  |  |
| IL-2 | -0.47* | 0.17 | -0.15 | -0.28 | -0.22 | -0.18 | -0.29 | 0.14 | 0.20 |
| IFNγ | -0.21 | 0.03 | 0.08 | -0.08 | 0.19 | -0.02 | -0.20 | 0.07 | 0.46* |
| TNFα | -0.38 | 0.11 | -0.04 | -0.14 | -0.06 | -0.01 | -0.34 | 0.01 | 0.29 |
| Th2 cell-related |  |  |  |  |  |  |  |  |  |
| IL-4 | -0.28 | 0.16 | 0.03 | -0.17 | 0.02 | -0.05 | -0.13 | 0.25 | 0.16 |
| IL-5 | -0.19 | 0.24 | -0.27 | -0.31 | -0.06 | -0.13 | -0.39* | 0.11 | 0.27 |
| IL-6 | -0.22 | 0.08 | 0.09 | -0.16 | -0.15 | -0.07 | -0.20 | 0.19 | 0.22 |
| IL-10 | -0.15 | -0.20 | 0.23 | 0.09 | 0.12 | 0.14 | 0.26 | 0.67* | -0.19 |
| IL-13 | -0.19 | 0.19 | -0.03 | -0.21 | -0.02 | -0.01 | -0.28 | 0.34 | 0.09 |
| Th17 cell-related |  |  |  |  |  |  |  |  |  |
| IL-1β | -0.29 | 0.38 | -0.13 | -0.48* | -0.12 | -0.17 | -0.51* | -0.12 | 0.34 |
| IL-17A | -0.29 | 0.13 | -0.12 | -0.21 | 0.08 | -0.12 | -0.30 | 0.10 | 0.32 |
| IL-23 | 0.01 | 0.03 | 0.01 | 0.17 | 0.20 | 0.26 | 0.04 | -0.05 | -0.13 |
| IL-25 | 0.09 | 0.12 | -0.13 | -0.03 | 0.06 | 0.32 | -0.36 | 0.52* | -0.21 |
| IL-31 | -0.11 | 0.40* | -0.41* | -0.34 | -0.40* | -0.05 | -0.46* | -0.05 | 0.00 |
| sCD40L |  |  |  |  |  |  |  |  |  |
| CXC chemokine | -0.25 | 0.24 | -0.13 | -0.23 | -0.09 | -0.01 | -0.33 | 0.20 | 0.10 |
| IL-8 | -0.02 | -0.19 | 0.16 | 0.24 | -0.07 | 0.10 | -0.06 | 0.27 | 0.05 |
| IP10 |  |  |  |  |  |  |  |  |  |
| CC chemokine | -0.17 | -0.02 | 0.15 | 0.05 | -0.04 | 0.08 | 0.11 | 0.50* | -0.23 |
| MCP1 | -0.04 | 0.23 | -0.13 | -0.19 | -0.02 | -0.10 | -0.52* | -0.28 | 0.45* |
| MIP1α | -0.02 | -0.21 | 0.26 | 0.25 | 0.08 | 0.10 | 0.01 | 0.25 | -0.12 |
| MIP1β | 0.03 | -0.29 | 0.25 | 0.38 | 0.00 | 0.14 | -0.01 | 0.42* | -0.17 |
| RANTES | -0.10 | 0.03 | 0.14 | 0.00 | 0.13 | 0.10 | -0.07 | 0.36 | 0.03 |
| Eotaxin |  |  |  |  |  |  |  |  |  |
| Others | -0.18 | 0.23 | -0.15 | -0.21 | -0.16 | -0.17 | -0.22 | -0.24 | 0.32 |
| IL-1RA | -0.30 | 0.26 | -0.04 | -0.14 | 0.06 | -0.03 | -0.27 | 0.10 | 0.19 |
| IL-7 | -0.06 | -0.25 | -0.01 | 0.19 | -0.15 | 0.05 | -0.08 | 0.22 | -0.12 |
| IL-9 | -0.20 | 0.12 | -0.12 | -0.12 | 0.00 | -0.08 | -0.44* | 0.11 | 0.23 |
| FGF | -0.31 | 0.24 | -0.05 | -0.26 | -0.01 | -0.10 | -0.29 | 0.17 | 0.22 |
| GCSF | 0.75* | -0.50 | 0.15 | 0.38 | 0.19 | 0.23 | 0.15 | 0.02 | -0.14 |
| GM-CSF | -0.29 | -0.03 | -0.12 | -0.10 | -0.40* | -0.17 | -0.14 | 0.15 | -0.18 |
| PDGF-BB | -0.07 | -0.04 | -0.15 | -0.02 | -0.06 | -0.15 | -0.04 | -0.02 | 0.31 |
| VEGF | -0.19 | -0.05 | -0.08 | 0.03 | -0.35 | -0.10 | -0.14 | 0.09 | -0.10 |

^Abbreviations: FGF, fibroblast growth factor; GCSF, granulocyte colony-stimulating factor; GM-CSF, granulocyte-macrophage colony-stimulating factor; IFNγ; interferon-gamma; IL, interleukin; IL-1RA, IL-1 receptor antagonist; IP10, IFNγ-induced protein 10; MCI, mild cognitive impairment; MCP1; monocyte chemoattractant protein 1; MIP1α, macrophage inflammatory protein 1-alpha; PDGF-BB, platelet-derived growth factor-BB; RANTES, regulated upon activation, normal T cell expressed and secreted; sCD40L, soluble CD40 ligand; Th, T helper; TNFα, tumor necrosis factor-alpha; and VEGF, vascular endothelial growth factor;^

* *P* value <0.05.
